# An ArcA-Modulated Small RNA in Pathogenic *Escherichia coli* K1

**DOI:** 10.3389/fmicb.2020.574833

**Published:** 2020-11-23

**Authors:** Hao Sun, Yajun Song, Fang Chen, Changhong Zhou, Peng Liu, Yu Fan, Yangyang Zheng, Xuehua Wan, Lu Feng

**Affiliations:** ^1^TEDA Institute of Biological Sciences and Biotechnology, Nankai University, Tianjin, China; ^2^The Key Laboratory of Molecular Microbiology and Technology, Ministry of Education, Nankai University, Tianjin, China; ^3^Tianjin Key Laboratory of Microbial Functional Genomics, Tianjin, China; ^4^College of Life Sciences, Nankai University, Tianjin, China

**Keywords:** *Escherichia coli* K1, meningitis, ArcA, small RNA, oxygen

## Abstract

*Escherichia coli* K1 is the leading cause of meningitis in newborns. Understanding the molecular basis of *E. coli* K1 pathogenicity will help develop treatment of meningitis and prevent neurological sequelae. *E. coli* K1 replicates in host blood and forms a high level of bacteremia to cause meningitis in human. However, the mechanisms that *E. coli* K1 employs to sense niche signals for survival in host blood are poorly understood. We identified one intergenic region in *E. coli* K1 genome that encodes a novel small RNA, sRNA-17. The expression of sRNA-17 was downregulated by ArcA in microaerophilic blood. The Δ*sRNA-17* strain grew better in blood than did the wild-type strain and enhanced invasion frequency in human brain microvascular endothelial cells. Transcriptome analyses revealed that sRNA-17 regulates tens of differentially expressed genes. These data indicate that ArcA downregulates the *sRNA-17* expression to benefit bacterial survival in blood and penetration of the blood–brain barrier. Our findings reveal a signaling mechanism in *E. coli* K1 for host adaptation.

## Introduction

Invasion of the central nervous system (CNS) by pathogenic bacteria causes meningitis, which results in 170,000 deaths worldwide per year (World Health Organization [WHO], 2018). High mortality and morbidity are associated with bacterial meningitis. Despite the advances in the treatment of bacterial meningitis using antimicrobials such as ampicillin, chloramphenicol, ceftriaxone, cefotaxime, and vancomycin (Borchardt, 2004), most of patients survived from meningitis continue suffering from long-term neurological sequelae including functional and behavioral abilities.

*Escherichia coli* K1 is the most common gram-negative bacterium that causes meningitis in newborns (Kim, 2010; Ouchenir et al., 2017). To cross the blood–brain barrier (BBB) and invade CNS, *E. coli* K1 has to escape host defense system, survive and replicate in the blood, and form a high level of bacteremia (Doran et al., 2016). Previous studies have identified that K1 capsular polysaccharide and O-lipopolysaccharide (LPS) play critical roles in induction of a high degree of bacteremia. *E. coli* NlpI and OmpA also contribute to bacteremia (Prasadarao, 2002; Tseng et al., 2012). However, additional *E. coli* K1 factors that contribute to the high level of bacteremia and the sensing-signaling mechanisms that *E. coli* K1 employs for its survival in host blood are not fully understood yet.

To survive and proliferate in host, pathogenic bacteria have evolved global regulatory systems to detect niche signals for controlling virulence gene expressions in host. Conditions such as oxygen and nutrient availabilities vary among specific host sites. Oxygen in host sites can serve as a niche signal exploited by pathogens for colonization and survival (Wan et al., 2009; Melson and Kendall, 2019). As a central regulatory system, the two-component system ArcA–ArcB senses oxygen deprivation and regulates expressions of a large number of genes for anaerobic adaptation to host sites (Iuchi and Lin, 1988; Liu and De Wulf, 2004; Deng et al., 2013; Fernández et al., 2018; Pardo-Esté et al., 2019). Moreover, ArcA controls bacterial resistance to reactive oxygen and nitrogen species (ROS and RNS) (Lu et al., 2002). In *Haemophilus influenzae*, upon exposure to human blood (a microaerophilic condition compared with air), the *arcA* mutants showed markedly reduced survival rates compared with those of the wild-type strain (De Souza-Hart et al., 2003). These data suggest a more complicated role of ArcA than what we currently understood. Bioinformatic analyses identified a common sequence recognition element consisting of two direct repeat elements with various spacing lengths (GTTA{x}_n_GTTA, x = A/T/G/C) (Park et al., 2013). Further analysis of ArcA-regulated genes will help understand its role in bacterial pathogenicity comprehensively.

Small RNAs are ubiquitous regulators in all three kingdoms of life. Bacteria employ small RNAs ranging from 50 to 500 nucleotides for posttranscriptional regulation (Dydecka et al., 2017). These sRNAs fold into diverse structures and act on downstream gene targets with different molecular mechanisms. A bacterial single sRNA can play a central regulatory role in controlling multiple gene targets leading to a global regulation effect (Modi et al., 2011; Gerrick et al., 2018; Gaimster et al., 2019). By this way, bacteria adapt to environmental stress quickly by regulating posttranscriptional responses. Despite their importance, the roles of sRNAs in the pathogenicity of *E. coli* K1 are poorly understood.

In this work, we used bioinformatic analysis to survey the potential binding sites of ArcA in the intergenic regions in the *E. coli* K1 Rs218 genome. We identified a small RNA, sRNA-17, which is under the control of ArcA regulation *in vitro*. Our findings reveal a central signaling mechanism employed by *E. coli* K1 for its adaptation to mammalian host.

## Materials and Methods

### Ethics Statement

All animal experiments were conducted in accordance with the criteria specified in the Guide for the Nursing and Use of Laboratory Animals. The animal research procedures were approved by the Institutional Animal Care Committee at Nankai University and Tianjin Institute of Pharmaceutical Research New Drug Evaluation (IACUC 2016032102), Tianjin, China. Every effort was made to minimize animal suffering and to reduce the number of animals used.

### Bacterial Strains, Plasmids, and Growth Conditions

Bacterial strains and plasmids used in this study are listed in Supplementary Table S1. Oligonucleotide primers used in this study are listed in Supplementary Table S2. *Escherichia coli* K1 Rs218 was used as the wild-type strain throughout this study. Mutant strains were generated by the λ Red recombinase system of pKD3, using primers carrying 39-bp homologous regions flanking the start and stop codons of the gene to be deleted, as previously described (Juhas and Ajioka, 2016). The plasmid for overexpression of sRNA-17 in Δ*sRNA-17* mutant was generated by cloning the *sRNA-17* sequence to a high copy number vector pBluescript II SK(+). All the strains were grown at 37°C in Luria–Bertani (LB) medium. Cultures were grown in LB medium with a loose cap aerobically (with shaking at 250 rpm) or microaerophilically (stationary growth), as previously described (Fan et al., 2014; Jiang et al., 2017). Antibiotics and chemicals were used at the following concentrations when necessary: ampicillin, 100 μg/ml; chloramphenicol, 25 μg/ml; and kanamycin, 50 μg/ml.

### *Escherichia coli* Invasion Assay in Human Brain Microvascular Endothelial Cell

Human brain microvascular endothelial cells (HBMECs) were a generous gift from Dr. K. S. Kim (Johns Hopkins University, Baltimore, MD, United States). The immortalized HBMECs have been verified for the function and morphological structure, which are similar to primary cells (Stins et al., 2001). HBMECs were cultured in Dulbecco’s modified Eagle’s medium (DMEM) with 10% fetal bovine serum, 10% Nu-serum (BD Biosciences), 2 mM of glutamine, and 1 mM of pyruvate. The Rs218 strain was grown overnight in LB medium and 1:100 subcultured into fresh LB medium for about 2 h until the strain was grown in exponential phase in OD_600_ of 0.6. The strain was collected by centrifugation at 5,000 rpm for 5 min and resuspended in experimental medium [M199-HamF12 (1:1)], containing 5% heat-inactivated fetal bovine serum, 2 mM of glutamine, and 1 mM of pyruvate (Zhu et al., 2010). The multiplicity of infection was 100:1. HBMECs and the strain were incubated at 37°C in a 5% CO_2_ incubator for 90 min. The monolayers were then washed with phosphate-buffered saline (PBS) and incubated with experimental medium containing gentamicin (100 mg/ml) for 1 h at 37°C to kill extracellular bacteria. HBMECs were washed, lysed with 0.5% Triton X-100 in PBS, and cultured for determination of colony-forming units (CFU). All invasion experiments were conducted in duplicate and performed a minimum of three times.

### Animal Model of *Escherichia coli* Bacteremia

Approximately 18-day-old BALB/c mice were used for bacteremia study. Each mouse received *E. coli* Rs218 (1 × 10^7^ CFU) at exponential phase in 100 μl of PBS via the tail vein injection. Four hours later, blood specimens were collected for determination of CFU and RNA extraction. For determination of CFU, bacteria in blood specimens were subjected to serial 10-fold dilutions in PBS and enumerated by plating on LB agar plates. For RNA extraction, mice were sacrificed, and blood specimens were collected to extract RNA using TRIzol reagent (Invitrogen, United States). Bacterial RNA was enriched using MICROBEnrich^TM^ (Invitrogen, United States).

### Mouse Model of Hematogenous Meningitis

Approximately 18-day-old BALB/c mice were used for hematogenous meningitis study. Each mouse received *E. coli* Rs218 (5 × 10^5^ CFU) at exponential phase in 100 μl of PBS via the tail vein injection. Four hours later, blood and cerebrospinal fluid (CSF) specimens were collected for determination of CFU.

### Yeast Aggregation Assay

The capacity of *E. coli* to agglutinate yeast cells was tested (Eshdat et al., 1981; Teng et al., 2005). Briefly, overnight cultures grown in LB medium were diluted 1:100 in LB medium and grown at 37°C with agitation until the OD_600_ was 0.600; 0.5 ml of bacteria under OD_600_ = 0.600 was centrifuged at 5,000 rpm/min, and the supernatant was removed. Commercial baker’s yeast cells (5 mg/ml) were added to bacteria. The mixture was resuspended and then incubated at room temperature for 10 min and then centrifuged at 750 × *g* to remove the yeast cells and their aggregates. The bacteria in the supernatant were counted. The aggregation rate was expressed as (1 − number of CFU per ml of bacteria under OD_600_ = 0.600/number of CFU per supernatant after aggregation) × 100%.

### 5′/3′ Rapid Amplification of Complementary DNA Ends

Simultaneous determination of 5′ and 3′ ends of sRNA-17 was performed using rapid amplification of complementary DNA ends (RACE) essentially as described (Toledo-Arana et al., 2009). The oligonucleotide primers used are listed in Supplementary Table S2. In each case, the generated PCR products were directly cloned into the pEASY-T1 vector for TA clone. Plasmids with sRNA RACE inserts were sequenced using M13 forward and reverse primers on an ABI 3730XL DNA sequencer at BGI.

### Northern Blot Analysis

Total RNA of about 10 μg was treated with DNase I and separated on a 1.2% formaldehyde-deformed agarose gel. RNA was transferred to nitrocellulose membranes and fixed for 30 min at 120°C. DIG Oligonucleotide Tailing Kit was used for digoxigenin tailing. DIG-probes (10 pM) were used for hybridization at 50°C overnight after the membrane was pre-hybridized in hybridization buffer for 30 min. Membranes were washed [2 × 5 min in 2 × SSC, 0.1% sodium dodecyl sulfate (SDS); 2 × 15 min in 0.1 × SSC, 0.1% SDS] and blocked in blocking solution for 30 min, incubated with antibody solution for another 30 min, and washed twice for 15 min. The membranes were balanced for 2–5 min in detection buffer and detected using CSPD-star and autoradiography.

### Quantitative Real-Time PCR

Quantitative real-time PCR (qRT-PCR) was performed using a 7500 Real-Time PCR system (Applied Biosystems). *E. coli* K1 Rs218, Δ*sRNA-17*, and Δ*arcA* strains were cultured overnight in triplicates and subsequently 1:100 subcultured in fresh LB medium to exponential phase. To analyze expressions of sRNA-17 at different growth phases, bacteria were harvested when growth reached OD_600_ of 0.4, 0.6, 1.0, and 1.8. Bacteria were pelleted by centrifugation. RNA samples were isolated using TRIzol (Invitrogen), reverse transcribed using PrimeScript^TM^ RT reagent Kit (Takara), and processed for qRT-PCR. Each qRT-PCR was carried out in a total volume of 20 μl in a 96-well optical reaction plate (Applied Biosystems) containing 10 μl of Power SYBR^TM^ Green PCR Master Mix (Applied Biosystems), 1 μl of cDNA, and two gene-specific primers with a final concentration of 0.3 μM each. The fold change in target gene relative to the housekeeping gene (16S rRNA) was determined by the 2^–ΔΔCt^ method. At least three biological replicates were performed for each qRT-PCR analysis.

### Protein Expression and Purification

For protein expression and purification, *arcA* was cloned into the pET28a with an N-terminal hexahistidine tag. His-tagged ArcA was overexpressed in *E. coli* BL21 cells induced with 0.1 mM of isopropyl-β-D-thiogalactopyranoside (IPTG) at 37°C for 3 h and purified by HiTrap Ni^2+^ chelating column. Purified proteins were analyzed by SDS–polyacrylamide gel electrophoresis (PAGE), and the concentration was determined by the Bradford protein assay (Bio-Rad) using a bovine serum albumin standard (Bradford, 1976). Aliquots of the purified protein were stored at −70°C.

### Electrophoretic Mobility Shift Assay

A DNA fragment containing 300 bp of the upstream region of sRNA-17 was amplified using primers sRNA-17F and sRNA-17R. Binding reactions between the DNA fragment and various amounts of purified his-tagged ArcA were performed in a solution containing 10 mM of Tris–HCl pH 7.4, 50 mM of KCl, 0.1 mM of EDTA, and 0.2 mM of DTT with or without 30 mM of acetyl phosphate. After incubation at 37°C for 30 min, reaction mixtures were then subjected to electrophoresis on a 6% polyacrylamide gel in 0.5 × TBE buffer (44.5 mM of Tris, 44.5 mM of boric acid, 1 mM of EDTA, pH 8.0) at 90 V for 90 min. The gel was stained in 0.5 × TBE buffer containing GelRed nucleic acid staining solution for 10 min.

### RNA-seq Analyses

Total RNA of wild-type Rs218 and Δ*sRNA-17* strains was isolated using TransZol Up Plus RNA kit (TransGen Biotech). Ribosomal RNAs were removed using Ribo-Zero rRNA removal kit (Illumina). RNA libraries were generated using NEB Next Ultra RNA Library Prep Kit (Illumina) and sequenced on an Illumina HiSeq 2000 platform. Quality control and filtering of raw sequences were carried out using FastQC^1^ and in-house perl program (NGQC, Novogene). The filtered reads were mapped to the reference genome of *E. coli* K1 Rs218 (GenBank accession numbers: CP007149 and CP007150). Differential gene expressions were analyzed using DEGseq (Wang et al., 2010). RNA-seq data have been submitted to the National Center for Biotechnology Information (NCBI) Sequence Read Archive (SRA) database under accession number PRJNA575116.

### Prediction of ArcA-Binding Site

Intergenic sequences were extracted from the genome sequence of *E. coli* Rs218 (GenBank accession numbers: CP007149 and CP007150) using samtools (Li et al., 2009). To identify intergenic regions that bind ArcA and encode potential small RNA, intergenic sequences above 1 kb were extracted. Both GTTA and its reverse complement sequence TAAC were searched in these intergenic regions. Intergenic regions were considered for BPROM analysis (Solovyev and Salamov, 2011) and downstream qRT-PCR, when the center to center distances between two DR elements are 14∼20 bp.

### Bioinformatic Analyses

The secondary structure of sRNA-17 was predicted using Mfold program^2^. PHAge Search Tool (PHAST) (Zhou et al., 2011) was used to identify prophage sequences in the *E. coli* Rs218 genome. Comparison of the genomes of *E. coli* Rs218 and *E. coli* K12 MG1655 (GenBank accession number: CP025268) was performed using Mauve software (Rissman et al., 2009), showing that the prophage genome of *Enterobacteria* phage mEp460 is absent from the *E. coli* K12 MG1655 genome.

To identify the phylogenetic groups of *E. coli* strains, the presence and absence of *chuA*, *yjaA*, and TspE4.C2 genes in the complete genomes were analyzed using BLASTN (Clermont et al., 2000). The *chuA* gene (NCBI GenBank: QKP92252.1), *yjaA* gene (NCBI Gene ID: 948515), and TspE4.C2 (NCBI GenBank: AF222188.1) were used for analysis.

To evaluate whether sRNA-17 is expressed in *E. coli* strains, we downloaded 47 transcriptome datasets sequenced for *E. coli* K1 (seven datasets), APEC (six datasets), and UPEC (34 datasets). The reads were mapped to sRNA-17 gene using bowite2 (Langmead and Salzberg, 2012). sRNA-17 is expressed in one dataset of *E. coli* K1, six datasets of APCE, and seven datasets of UPEC (Supplementary Table S6).

### Statistical Analysis

Statistical analyses were performed using statistical analysis in social science program (SSPS) or R packages. Statistical significance between two groups was analyzed using Student’s *t*-test. When comparing multiple groups, we used one-way analysis of variance (ANOVA) or two-way ANOVA. *P* value of <0.05 was considered significant.

## Results

### Prediction of ArcA-Binding Sites in the *Escherichia coli* Rs218 Genome

**FIGURE 1:**
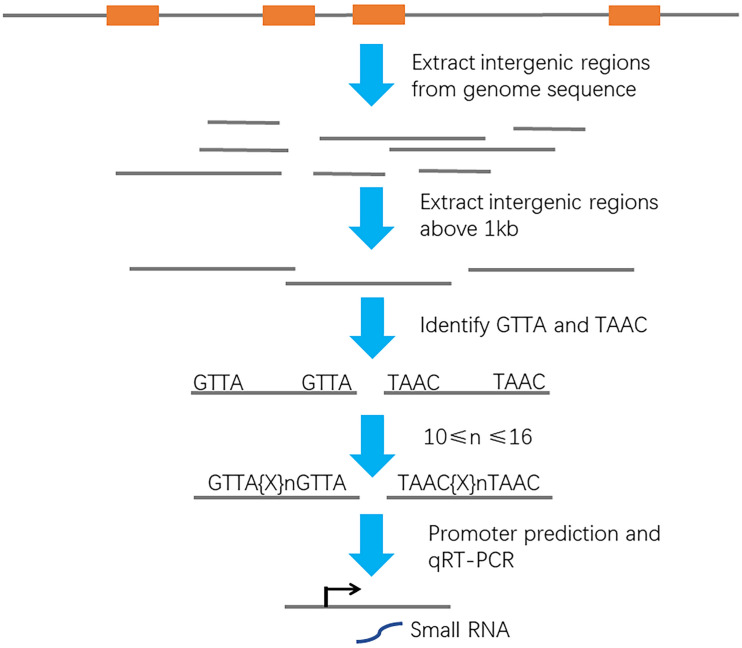
Schematic of a bioinformatic screen setup to identify ArcA-binding promoters. Orange box, protein-encoding gene. Gray line, intergenic region. Black arrow, transcription start site.

As a global regulator, ArcA regulates expressions of more than hundreds of protein-encoding genes at transcriptional level. Small non-coding RNAs under the ArcA regulation, which can degrade mRNA targets and regulate expressions of protein-encoding genes at transcriptional or posttranscriptional level, have been overlooked for decades. To search for ArcA regulated non-coding RNAs in meningitis-causing *Escherichia coli*, a genome-wide screen was performed (Figure 1). We downloaded the genome sequence of *E. coli* Rs218 (GenBank accession numbers: CP007149 and CP007150) from the NCBI database. The sequenced Rs218 strain (O18:H7:K1, ST95) was isolated from the CSF of a patient with neonatal meningitis in 1974 (Silver et al., 1980; Day et al., 2015; Wijetunge et al., 2015). We removed the genes annotated as hypothetical proteins from the annotation file and extracted 4,455 intergenic regions from the genome sequence. Next, we obtained the intergenic sequences whose lengths are above 1 kb and systematically screened them for the pattern of ArcA-binding site, GTTA{x}_n_GTTA (x = A/T/G/C). We found that 42 intergenic sequences above 1 kb contain potential ArcA-binding site. The BPROM program further identified ArcA-binding sites and promoter sites in 15 intergenic sequences (Solovyev and Salamov, 2011). Next, we carried out qRT-PCR to search for small RNA candidates. We found that one intergenic region (genome location, 4534659–4535810) may express a non-coding small RNA.

### Identification of a Novel Small RNA in *Escherichia coli* K1

**FIGURE 2:**
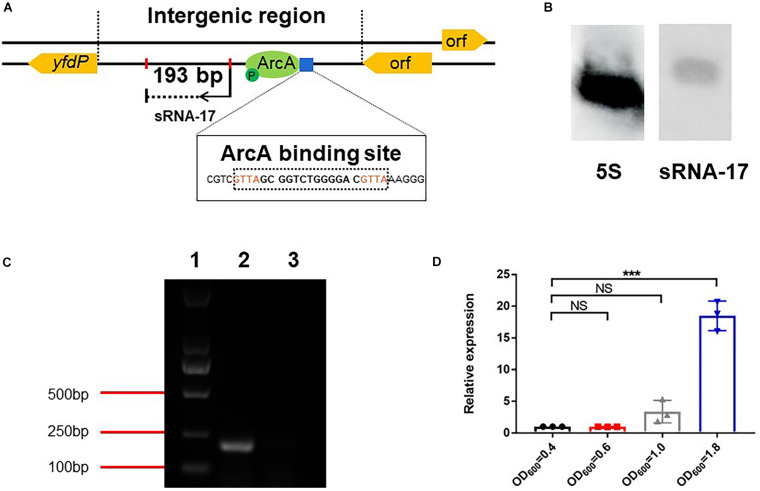
Expression analysis of sRNA-17. **(A)** Identification of sRNA-17 and ArcA-binding site in *Escherichia coli* K1 genome. **(B)** Northern blots were probed for sRNA-17 to further verify that sRNA-17 was a non-coding RNA. The 5S rRNA served as the loading control. **(C)** Electrophoresis of amplified sRNA-17 on an agarose gel. The primers used for amplification of sRNA-17 were designed based on rapid amplification of complementary DNA ends (RACE) sequencing result. Lane 1, marker D2000; lane 2, wild-type strain; lane 3, Δ*sRNA-17* strain. **(D)** Expression of sRNA-17 at different growth phases. Fold change in the RNA levels of sRNA-17 in the Rs218 strain when growth reached OD_600_ of 0.4, 0.6, 1.0, and 1.8, respectively. ***P < 0.001; NS, no significance, *P* > 0.05.

To determine the accurate 5′ and 3′ ends of the sRNA, we carried out RACE of both ends. 5′ and 3′ RACE identified a 193-bp sRNA. BLASTN analysis aligned the sRNA sequence to the negative strand of the *E. coli* Rs218 genome and confirmed that the small RNA locates in the intergenic region (genome location, 4535226–4535418) of the *E. coli* Rs218 genome. In addition, no homolog of the small RNA can be identified in the Rfam database, suggesting that it is a novel sRNA. We named the small RNA as sRNA-17 (Figure 2A). To confirm the expression of sRNA-17, we grew the Rs218 strain in LB medium at 37°C until the culture reached an OD_600_ of 0.5∼0.6. Northern blot analysis showed that sRNA-17 was expressed in this condition. Compared with the high expression level of 5S ribosomal RNA, the expression level of sRNA-17 was moderate (Figure 2B). The size of sRNA-17 was confirmed by electrophoresis of the amplified product of cDNA on an agarose gel (Figure 2C). We next compared expression levels of sRNA-17 in the Rs218 strain grown at exponential and stationary phases. Figure 2D shows that expression of sRNA-17 dramatically increased in the Rs218 strain at stationary phase. A secondary structure of sRNA-17 was predicted using Mfold program as shown in Supplementary Figure 1.

### Oxygen-Dependent Expression of sRNA-17

**FIGURE 3:**
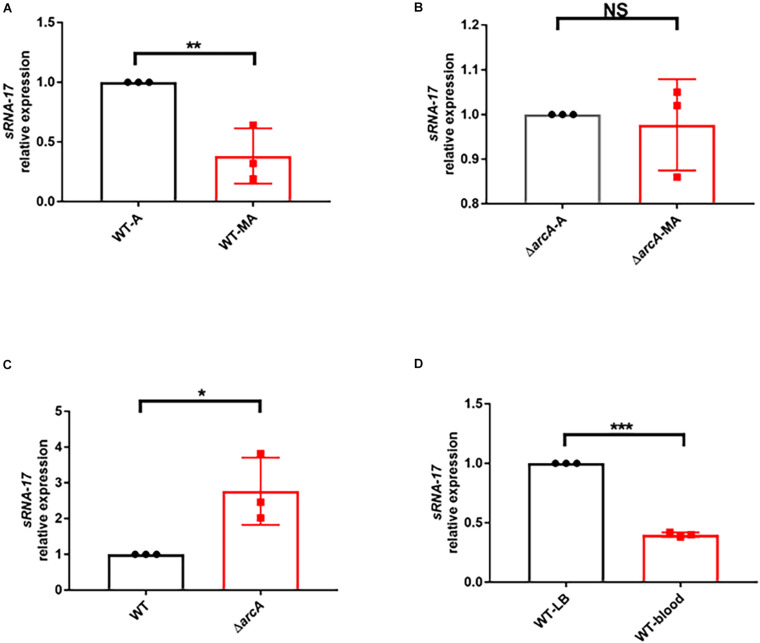
The expression of sRNA-17 in RNA level by real-time PCR. **(A)** Fold change in the RNA levels of sRNA-17 in the wild-type strain in microaerophilic (WT-MA) and aerobic (WT-A) conditions. **(B)** Fold change in the RNA levels of sRNA-17 in Δ*arcA* strain in microaerophilic (Δ*arcA*-MA) and aerobic (Δ*arcA*-A) conditions. **(C)** Fold change in the RNA levels of sRNA-17 in the wild-type and Δ*arcA* strains. **(D)** Fold change in the RNA levels of sRNA-17 in Luria–Bertani (LB) medium and mouse blood by intravenous injection via the tail vein. *P < 0.05; **P < 0.01; ***P < 0.001; NS, no significance, *P* > 0.05.

Because the above bioinformatic analysis predicted the ArcA-binding site in the promoter region of sRNA-17, one would expect to see that expression of sRNA-17 is oxygen dependent. To investigate this hypothesis, we cultured Rs218 strain in LB medium at 37°C aerobically (with shaking at 250 rpm) and microaerophilically (stationary growth), according to the previous method (Fan et al., 2014; Jiang et al., 2017). qRT-PCR detected the expression levels of sRNA-17 in these two conditions (Figure 3A). The expression level of sRNA-17 was 2.6-fold lower in a microaerophilic condition than that in an aerobic condition (Figure 3A), confirming that expression of sRNA-17 in *E. coli* Rs218 strain is oxygen dependent. Next, we generated an in-frame Δ*arcA* strain constructed by replacing the coding region of *arcA* with a Cm^R^ resistance cassette flanked by FLP recognition target (FRT) sites (Juhas and Ajioka, 2016). By contrast, the expression levels of sRNA-17 in Δ*arcA* strain in microaerophilic and aerobic conditions showed no significant difference (Figure 3B). In addition, the expression level of sRNA-17 in Δ*arcA* strain was three-fold higher than that in wild-type strain (Figure 3C). Taken together, these data indicate that ArcA inhibits sRNA-17 expression in a low-oxygen condition.

Because the oxygen concentration in blood is lower than that in atmosphere, we would expect to see that the expression level of sRNA-17 in *E. coli* Rs218 grown in blood is lower than that in LB medium at 37°C with shaking at 250 rpm. To test this, we examined the expression level of sRNA-17 *in vivo*. Eighteen-day-old BALB/c mice received 1 × 10^7^ CFU of the Rs218 strain via tail vein injection. After 4 h, bacteria from blood were collected for total RNA isolation. The expression of sRNA-17 was 2.5-fold lower in bacteria from blood than that in bacteria grown in aerobic LB medium (Figure 3D). These data suggest that expression of sRNA-17 is downregulated in *E. coli* K1 in blood compared with that in aerobic LB medium.

### Phosphorylated ArcA Binds the Promoter of sRNA-17

**FIGURE 4:**
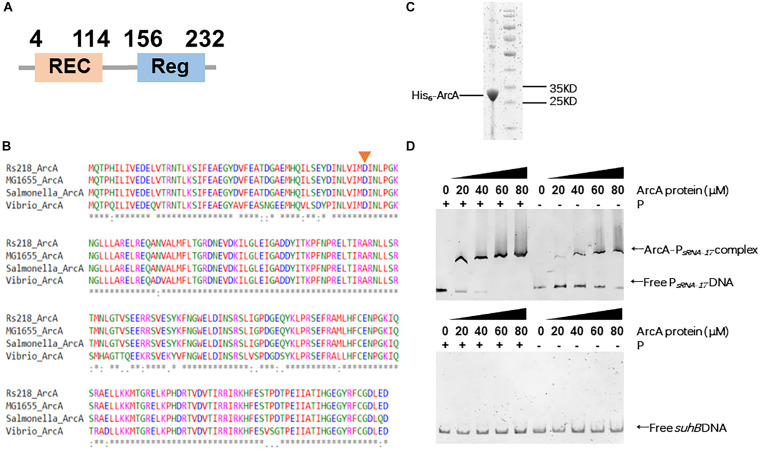
Phosphorylated ArcA strongly binds the sRNA-17 promoter from *Escherichia coli* K1 Rs218. **(A)** Domain organization of ArcA protein from *E. coli* K1 Rs218. **(B)** MAFFT-based protein sequence alignment of ArcA proteins from *E. coli* K1 Rs218, *E. coli* K12 MG1655, *Salmonella enterica* subsp. *enterica* serovar Typhimurium American Type Culture Collection (ATCC) 14028, and *Vibrio cholerae* O1 biovar EI Tor str. N16961. The aspartic acid residue 54 (indicated by an orange arrow), which receives phosphorelay is conserved in the four ArcA proteins. An * indicates that amino acid positions have a single, fully conserved residue. A: indicates amino acid positions are conserved with groups of strongly similar properties. **(C)** Sodium dodecyl sulfate–polyacrylamide gel electrophoresis (SDS-PAGE) of purified ArcA-His_6_ by HiTrap Ni^2+^-chelating column. **(D)** Electrophoretic mobility shift assays (EMSAs) of sRNA-17 promoter DNA fragment with purified ArcA-His_6_ protein (0, 20, 40, 60, and 80 μM) with or without the acetyl phosphate **(top panel)**. EMSA of *suhB* gene fragment with purified ArcA-His_6_ protein (0, 20, 40, 60, and 80 μM) with or without the acetyl phosphate **(bottom panel)**.

The SMART domain analysis of ArcA from the Rs218 strain confirmed that ArcA contains an N-terminal REC domain and a C-terminal DNA binding domain (Figure 4A). To examine the phosphorylation site at ArcA, we performed the alignment of ArcA proteins from Rs218, *E. coli* K12 MG1655, *Salmonella enterica* subsp. *enterica* serovar Typhimurium American Type Culture Collection (ATCC) 14028, and *Vibrio cholerae* O1 biovar EI Tor str. N16961, using MAFFT program. Figure 4B shows that the phosphorylation site (aspartic acid residue 54) was conserved in these ArcA proteins, suggesting that ArcA from *E. coli* Rs218 is functional. To confirm that ArcA binds the promoter of sRNA-17 and phosphorylated ArcA binds the promoter of sRNA-17 better, we purified the recombinant Rs218 ArcA protein containing an N-terminal hexahistidine tag from *E. coli* BL21 (Figure 4C). Then we carried out electrophoretic mobility shift assay (EMSA) to show that the phosphorylated ArcA (with presence of acetyl phosphate) bound the sRNA-17 promoter better than the unphosphorylated ArcA (Figure 4D). By contrast, EMSA showed that ArcA did not bind the amplified DNA fragment of *suhB* gene that served as the negative control (Figure 4D). These data suggest that phosphor-activated ArcA binds the sRNA-17 promoter strongly.

### Global Gene Expression Regulated via sRNA-17 in *Escherichia coli* Rs218

**FIGURE 5:**
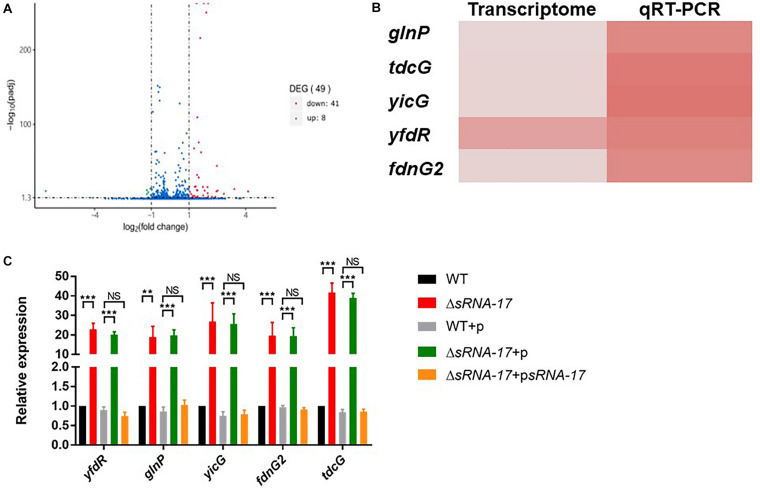
Transcriptome analysis reveals differentially expressed genes regulated by sRNA-17. **(A)** Volcano plot shows the fold change of genes (log2 scale) between Δ*sRNA-17* strain and wild-type Rs218 strain in Luria–Bertani (LB) medium. Green dots, downregulated genes (fold change <0.5); red dots, upregulated genes (fold change >2). See Table 1 and Supplementary Table S3 for upregulated and downregulated gene lists, respectively. Adjusted *P* values were determined by DEGseq (Wang et al., 2010) based on Benjamini–Hochberg method. **(B)** Heatmap comparison of transcriptome and qRT-PCR analyses of upregulated genes in Δ*sRNA-17* strain compared with wild-type Rs218 strain. **(C)** Bar plot of qRT-PCR analyses of upregulated genes in the wild-type, Δ*sRNA-17*, and complementation strains. **P < 0.01; ***P < 0.001; NS, no significance, *P* > 0.05.

To examine the downstream genes whose transcriptions are regulated by sRNA-17, we generated an in-frame Δ*sRNA-17* strain constructed by replacing the coding region of *sRNA-17* with a Cm^R^ resistance cassette flanked by FRT sites (Juhas and Ajioka, 2016). We cultured wild-type Rs218 and Δ*sRNA-17* strains in LB medium at 37°C until the culture reached an OD_600_ of 0.5∼0.6. Bacteria cultures were collected, and total RNA was isolated for transcriptome sequencing. In total, 5.6 and 4.5 million paired-end sequencing reads were generated on Illumina HiSeq 2000 for wild-type Rs218 and Δ*sRNA-17* strains, respectively. More than 98% of the quality trimmed reads for both samples were mapped to the Rs218 reference genome. Differential gene expression analysis revealed that seven genes were upregulated (fold change >2) and 41 genes were downregulated (fold change <0.5) in Δ*sRNA-17* strain compared with the wild-type Rs218 strain (Figure 5A and Table 1 and Supplementary Table S3). Because most of bacterial small RNAs function as *trans-*acting sRNAs, which degrade their mRNA targets (Gripenland et al., 2010), those upregulated genes in the absence of sRNA-17 can be the binding targets of sRNA-17. qRT-PCR analysis of the selected upregulated genes confirmed the transcriptome results (Figures 5B,C). Among these upregulated genes, *glnP*, two *fdnG*, and *tdcG* encode proteins involved in amino acid transport and metabolism. *glnP* encodes a glutamine transport system permease protein. *fdnG* encodes a formate dehydrogenase. *tdcG* encodes an anaerobically inducible I-serine dehydratase. These genes are involved in amino acid transport (*glnP*) and metabolism (*fdnG* and *tdcG*), which affect bacterial growth rate. These data suggest that suppression of sRNA-17 expression in *E. coli* Rs218 in blood enhances expressions of the genes involved in amino acid transport and metabolism, which benefit *E. coli* K1 growth in blood. Interestingly, a number of genes involved in protein folding (*groEL*) and fimbriae assembly (*fimACDFHI*) were downregulated in the Δ*sRNA-17* strain compared with the wild-type Rs218 strain (Supplementary Table S3), suggesting complex roles of the potential targets of sRNA-17.

**TABLE 1:** The upregulated genes in Δ*sRNA-17* compared with wild-type (WT) Rs218 strain.

Gene	WT_readcount	Δ*sRNA-17*_readcount	log2(Fold change)	*P* value
*glnP*	155.3129339	318.7590208	−1.0373	3.93E−16
*fdnG1*	61.78058069	125.6337112	−1.024	4.17E−07
*fdnG2*	92.0555266	214.4538233	−1.2201	5.03E−14
*tdcG*	20.42943505	44.9943989	−1.1391	0.0010397
*yicG*	81.47160242	180.269767	−1.1458	4.30E−11
*yfdR*	0	8.472971221	−4.0829	0.0034465
*yfdP*	0.492275543	47.03959885	−6.5783	8.33E−13

### Deletion of sRNA-17 Enhances *Escherichia coli* K1 Survival in Blood and Meningitis *in vivo*

**FIGURE 6:**
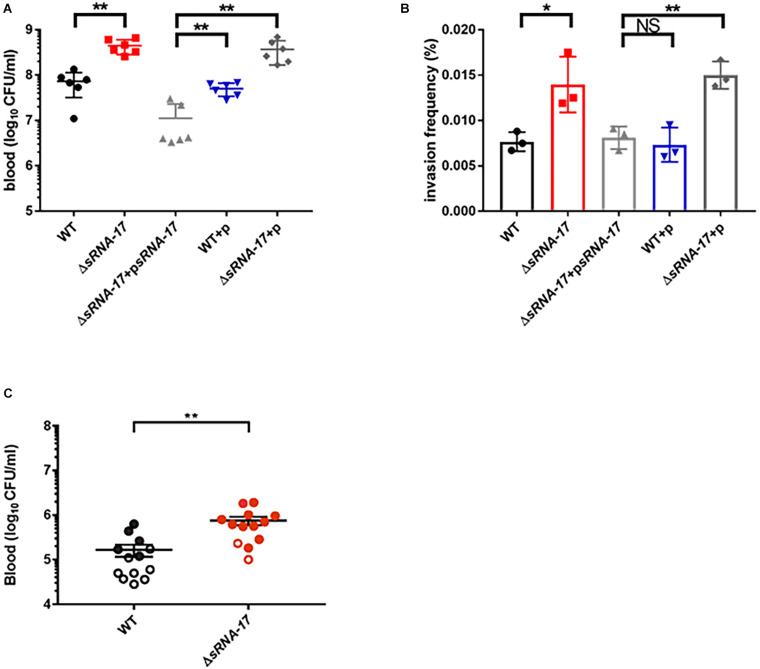
Loss of sRNA-17 enhances *Escherichia coli* K1 survival in blood and penetration of the human brain microvascular endothelial cells (HBMECs). **(A)** Bacterial counts in the blood [colony-forming units (CFU) per milliliter] were determined 4 h after intravenous injection of *E. coli* K1 strains via the tail vein. **(B)** The invasion frequency of Δ*sRNA-17* and overexpressed sRNA-17 strains compared with wild-type Rs218, wild-type Rs218 with vector, and Δ*sRNA-17* with vector strains. **(C)** The magnitude of bacteremia and the development of meningitis in BALB/c mice. Bacterial counts in the blood (CFU per milliliter) were determined 4 h after intravenous injection of *E. coli* K1 strains via the tail vein. Each circle represents bacterial counts in the blood of individual mouse. Black, the wild-type strain; red, Δ*sRNA-17* strain. The shaded or open circles represent the presence or absence of positive cerebrospinal fluid (CSF) cultures in each mouse. The data are means ± SD from three independent experiments. Each experiment was performed in triplicate. *P < 0.05; **P < 0.01; NS, no significance; *P* > 0.05 (Student’s *t*-test).

To investigate the role of sRNA-17 in *E. coli* K1 infection, we examined the abilities of wild-type Rs218 and Δ*sRNA-17* strains to survive in blood of BALB/c mice. Each animal received 1 × 10^7^ CFU of wild-type Rs218 and Δ*sRNA-17* strains via the tail vein injection. Four hours later, the blood specimens were collected for determination of CFU. Our data showed that Δ*sRNA-17* strain grew significantly better than the wild-type Rs218 strain *in vivo* (Figure 6A), suggesting that expression of sRNA-17 plays a role in inhibiting *E. coli* K1 proliferation in mouse blood. When sRNA-17 was highly expressed in Δ*sRNA-17* strain via pBluescript II SK(+) plasmid, the Δ*sRNA*-*17*-psRNA-17 strain grew less than the wild-type Rs218 strain with pBluescript II SK(+) vector (Figure 6A), confirming that expression of sRNA-17 inhibits *E. coli* K1 proliferation *in vivo*.

To cause meningitis, *E. coli* K1 proliferates in blood and penetrates across the BBB as live bacterium. To examine whether sRNA-17 is involved in regulating *E. coli* for penetrating the BBB, we carried out an HBMEC invasion assay to compare the invasion abilities of the wild-type Rs218 and Δ*sRNA-17* strains with a multiplicity of infection (MOI) of 100. Figure 6B shows that Δ*sRNA-17* strain entered HBMECs with an invasion frequency of 0.011 to 0.014%, which was 1.65-fold greater than the invasion frequency of wild-type Rs218 strain (0.0067 to 0.0088%, *P* < 0.05). The Δ*sRNA*-*17*-psRNA-17 strain entered HBMECs with an invasion frequency of 0.0067 to 0.0091%, which showed no significant difference from wild-type Rs218 strain carrying a pBluescript II SK(+) vector (Figure 6B). Because the above transcriptome analysis suggests that *fim* genes, which play an important role in cell adhesin, were downregulated in Δ*sRNA-17* strain, we next performed a yeast agglutination assay (Eshdat et al., 1981) to examine whether deletion of sRNA-17 affects the capacity of the Rs218 strain to agglutinate *Saccharomyces cerevisiae*. Supplementary Figure 2 shows that both wild-type and Δ*sRNA-17* strains attached to the yeast cells with the same abilities, indicating that the absence of sRNA-17 does not affect fimbriae activity in *E. coli* Rs218. Taken together, these data suggest that sRNA-17 regulates expression of genes involved in penetration of the BBB.

Next, we examined the effect of sRNA-17 on causing meningitis using a mouse model of hematogenous meningitis. When injected with the same CFU numbers (5 × 10^5^) of the wild-type and Δ*sRNA-17* strains, the development of *E. coli* meningitis (defined as positive CSF cultures) was significantly more in the recipients of Δ*sRNA-17* strain than in mice that received the wild-type strain (Figure 6C). These data indicate that decreased expression of sRNA-17 promotes meningitis *in vivo*.

### The Novel sRNA-17 Carried by Mobile Genetic Element Is Present in Other Genera and *Escherichia coli* Strains

Next, we asked whether sRNA-17 is present in the genera outside *Escherichia*. To examine this, we run BLASTN against the nucleotide collection database at NCBI website. Besides, homologs were also found in *S. enterica*, *Escherichia albertii*, *Escherichia fergusonii*, *Shigella dysenteriae*, and *Enterobacteria* phage mEp460, indicating that homologs of sRNA-17 are present in other genera outside *Escherichia*, which may be horizontally transferred via phage, a mobile genetic element. We next carried out PHAge Search Tool (PHAST) analysis of the Rs218 genome (Zhou et al., 2011) and confirmed that sRNA-17 locates in an intact prophage genome of *Enterobacteria* phage mEp460 that is absent from the genome of *E. coli* K12 MG1655. Taken together, sRNA-17 is present in other genera and may be horizontally transferred via phage between different genera and strains.

To examine whether sRNA-17 is ubiquitously present in *E. coli* strains, we downloaded 1,676 complete genomes of *E. coli* strains from the NCBI database. BLASTN analysis identified 60 *E. coli* genomes with the 193-bp sRNA-17 (Supplementary Table S4) and 125 *E. coli* genomes with the degenerative sRNA-17 (length <193 bp) (Supplementary Table S5 and Supplementary File), which were distributed in the four phylogenetic groups of *E. coli* including A, B1, B2, and D. Most of these *E. coli* strains are pathogenic *E. coli* including neonatal meningitis-causing *E. coli* (NMEC), uropathogenic pathogen *E. coli* (UPEC), avian pathogenic *E. coli* (APEC), enterotoxigenic *E. coli* (ETEC), Shiga toxin-producing *E. coli* (STEC), and unclassified pathogens (Supplementary Tables S4, S5). To examine whether sRNA-17 homologs are actively expressed in pathogenic *E. coli* strains, we searched the transcriptomic database at NCBI and mapped the downloaded reads of *E. coli* K1, APEC, and UPEC to sRNA-17 gene using bowtie2 (Langmead and Salzberg, 2012). Besides *E. coli* Rs218 (dataset in this study), sRNA-17 was found to be expressed in APEC when invading the mouse brain microvascular endothelial cell line bEnd.3, UPEC isolated from patients with urinary tract infections, and UPEC under nitrosative stress (Supplementary Table S6; Mehta et al., 2015; Sintsova et al., 2019; Wang et al., 2020).

## Discussion

Successful pathogens exploit specific strategies to invade, survive, proliferate, and spread in host. Bacteria sense host signals such as oxygen and redox potential in specific niche and respond by changing their own gene expression profiles. For example, enterohemorrhagic *Escherichia coli* O157:H7 (EHEC) controls global expression of virulence genes via an oxygen-responsive small RNA DicF for host colonization (Melson and Kendall, 2019). The whooping cough pathogen *Bordetella pertussis* expresses a globin-coupled sensor, *Bpe*GReg, which senses oxygen and regulates biofilm formation for directing *B. pertussis* to colonize or not (Wan et al., 2009). Our findings uncover a novel small RNA and its roles in meningitis-causing *E. coli*. In this work, we identified a novel sRNA, sRNA-17, in an *E. coli* K1 Rs218 strain that was isolated from meningitis patient. When forming bacteremia in blood circulation, *E. coli* K1 downregulated expression of sRNA-17 for its proliferation compared with those in LB broth. We further identified that this regulation of expression of sRNA-17 is under the control of the transcriptional regulator ArcA in the two-component system ArcAB. In addition, our data reveal that sRNA-17 is a central regulatory sRNA that controls expressions of multiple downstream gene targets. Importantly, our findings refine a signaling mechanism that meningitis-causing *E. coli* utilizes an oxygen-responsive sRNA for its survival and proliferation in blood.

Oxygen transportation and storage in tissues are necessary for multicellular organism survival. The oxygen partial pressure (pO_2_) is defined as a key component of the physiological state of a mammalian organ. In respiration, oxygen diffuses passively across the respiratory membrane from the alveolus to the capillary, leading to a declined pO_2_ in blood (80–100 mmHg in veins and 40 mmHg in arteries) compared with that in air (160 mmHg). During infection, pathogenic bacteria need to respond and survive in such an anaerobic environment. The two-component system ArcAB is highly conserved and plays pivotal roles in important pathogenic bacteria such as *Salmonella enterica* serovar Typhimurium, *S. enterica* serovar Enteritidis, *Vibrio cholerae*, and *Haemophilus influenzae* (Lu et al., 2002; De Souza-Hart et al., 2003; Sengupta et al., 2003; Morales et al., 2013). In *E. coli* K1 Rs218, ArcB contains a transmembrane domain followed by PAS, HisKA, HATPase, REC, and HPT domains. In bacteria, PAS domain-containing proteins bind heme, flavin, and a 4-hydroxycinnamyl chromophore to sense molecular oxygen, redox potential, and light, respectively (Gilles-Gonzalez et al., 1991; Bibikov et al., 2000; Tuckerman et al., 2009). ArcB in *E. coli* is a transmembrane protein that senses redox signal via the PAS domain to activate the phosphorelay at the C-terminal domains (Iuchi et al., 1990; Malpica et al., 2004). The cytosolic ArcA contains an N-terminal REC domain that can receive phosphor signal from ArcB and a C-terminal DNA binding domain that regulates gene transcription. Our EMSA result confirmed that the phosphorylation of ArcA enhanced the binding activity on the promoter of sRNA-17. Further studies will be necessary to confirm the redox signal in blood for activation of ArcB that leads to the repressed expression of sRNA-17.

**FIGURE 7:**
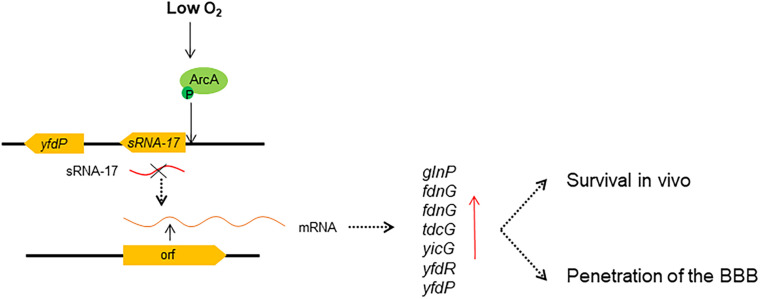
Regulatory model of ArcA and sRNA-17 in response to low oxygen signal. Our findings suggest that under a low-oxygen condition, phosphorylated ArcA binds the sRNA-17 promoter, which may inhibit the transcription of sRNA-17. When the sRNA-17 is absent, the differentially expressed genes benefit bacterial survival in blood and penetration of the blood–brain barrier (BBB). Red arrow, upregulated gene expression.

Our RNA-seq analysis revealed tens of differentially expressed genes in Δ*sRNA-17* strain compared with the wild-type Rs218 strain. The upregulated *glnP*, *fdnG*, and *tdcG* that are involved in amino acid transport and metabolism may benefit bacterial proliferation. In addition, the genes encoding membrane proteins may benefit *E. coli* K1 for crossing the BBB. Deletion of *glnP* gene in *Streptococcus pneumoniae* lowered the bacterial adhesion to Detroit 562 human pharyngeal epithelial cells (Kloosterman et al., 2006). Deletion of *glnP* gene in foodborne pathogen *Campylobacter jejuni* reduced its colonization in chicken host (de Vries et al., 2017). These data suggest that *glnP* gene plays an important role in pathogenic bacterial colonization to hosts. Thus, *E. coli* K1 senses redox signal in blood to downregulate expression of sRNA-17, leading to the upregulation of *glnP*, which benefits *E. coli* survival in blood and penetration of the BBB (see Figure 7 for schematic). However, these differential expressed genes may not be the direct targets of sRNA-17, and there may be other master regulatory proteins that serve as the target genes of sRNA-17 and regulate their expressions. Further screening of the specific direct targets of sRNA-17 will help us to understand the mechanism of ArcAB-regulated sRNA-17.

## Data Availability Statement

RNA-seq data have been submitted to the NCBI SRA database under accession number PRJNA575116.

## Ethics Statement

The animal study was reviewed and approved by the Institutional Animal Care Committee at Nankai University.

## Author Contributions

LF, XW, and HS conceived and designed the experiments, analyzed the data, and wrote the manuscript. HS, YS, FC, CZ, PL, YF, and YZ performed the experiments. All authors contributed to the article and approved the submitted version.

## Conflict of Interest

The authors declare that the research was conducted in the absence of any commercial or financial relationships that could be construed as a potential conflict of interest.

## Footnotes

1 https://www.bioinformatics.babraham.ac.uk/projects/fastqc/

2 http://unafold.rna.albany.edu/?q=mfold

## Supplementary Material

Supplementary Figure 1 A secondary structure of sRNA-17 predicted by Mfold.

Supplementary Figure 2 Agglutinating activities of the wild-type and Δ*sRNA-17* strains. NS, no significance, *P* > 0.05 (Student’s *t* test).

Supplementary File MAFFT-based sequence alignment of degenerative sRNA-17s and one 193-bp sRNA-17.
