# Supplementary figures and images for: An ArcA-Modulated Small RNA in Pathogenic Escherichia coli K1
Source: Front Microbiol. 2020 Nov 23;11:574833. doi: 10.3389/fmicb.2020.574833 (PMC7719688; doi:10.3389/fmicb.2020.574833)

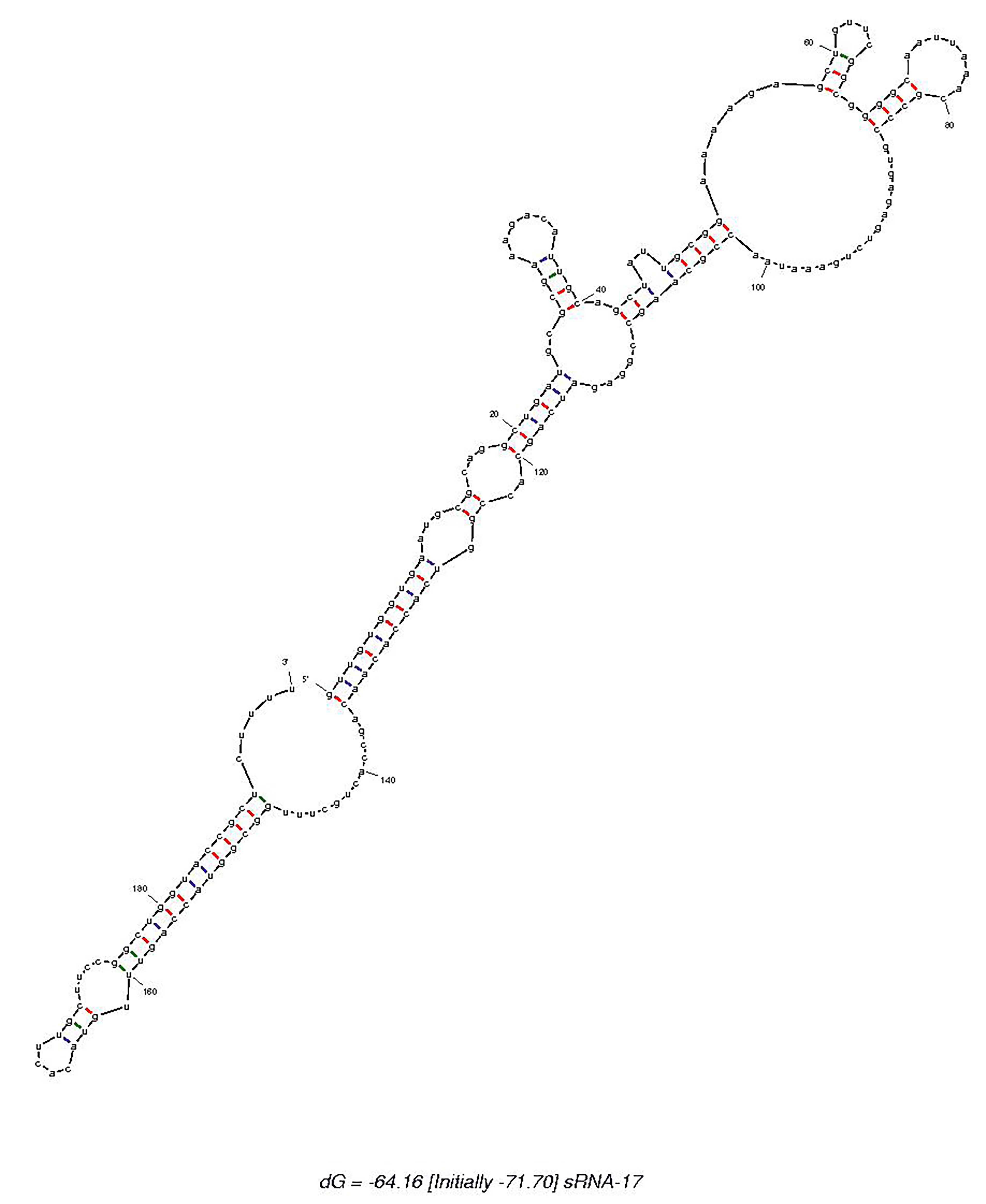

Supplement: Supplementary Figure 1 — A secondary structure of sRNA-17 predicted by Mfold. [file Image_1.JPEG]

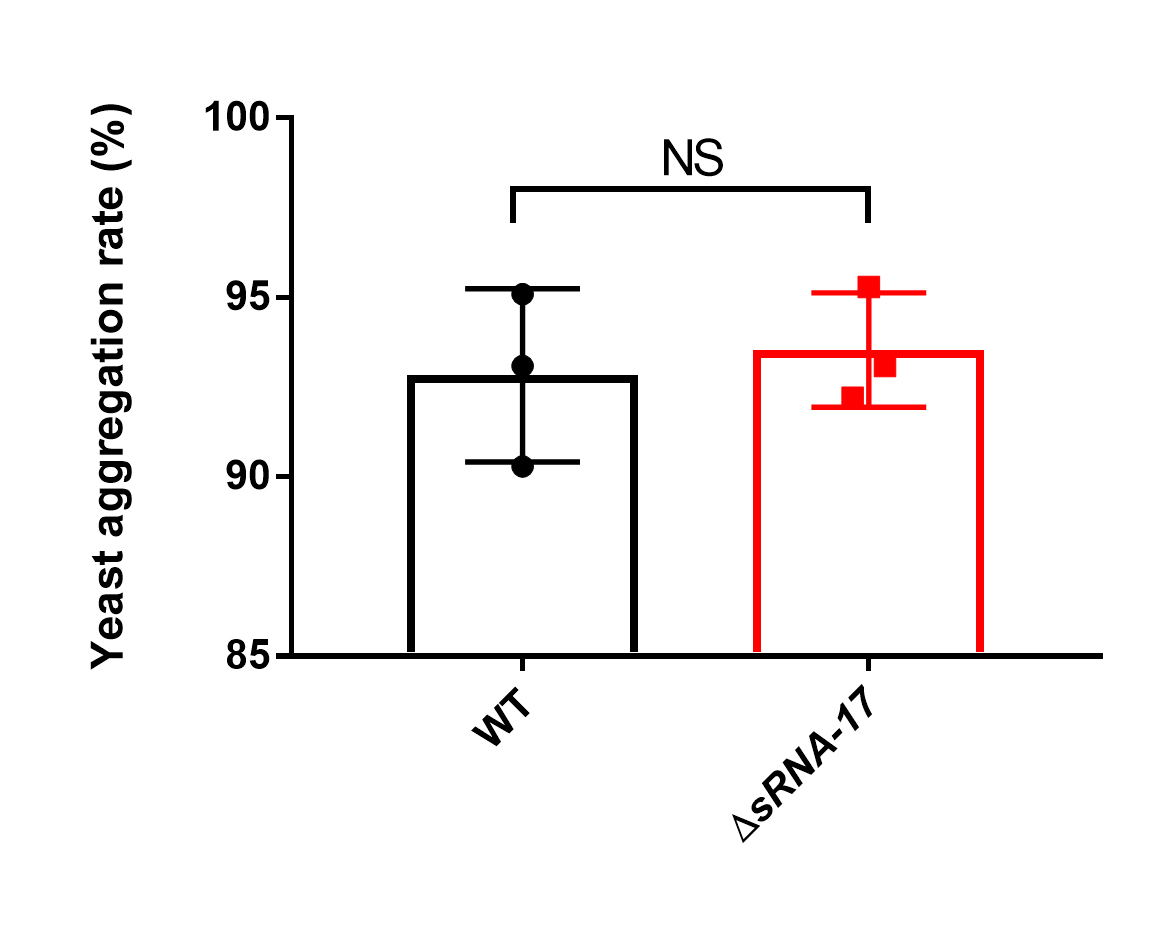

Supplement: Supplementary Figure 2 — Agglutinating activities of the wild-type and ΔsRNA-17 strains. NS, no significance, P > 0.05 (Student’s t test). [file Image_2.JPEG]
